# Positive Psychology: Looking Back and Looking Forward

**DOI:** 10.3389/fpsyg.2022.840062

**Published:** 2022-03-17

**Authors:** Carol D. Ryff

**Affiliations:** Department of Psychology, Institute on Aging, University of Wisconsin-Madison, Madison, WI, United States

**Keywords:** positive, negative, commercialization, inequality, greed, indifference, arts, nature

## Abstract

Envisioning the future of positive psychology (PP) requires looking at its past. To that end, I first review prior critiques of PP to underscore that certain early problems have persisted over time. I then selectively examine recent research to illustrate progress in certain areas as well as draw attention to recurrent problems. Key among them is promulgation of poorly constructed measures of well-being and reliance on homogeneous, privileged research samples. Another concern is the commercialization of PP, which points to the need for greater oversight and quality control in profit-seeking endeavors. Looking ahead, I advocate for future science tied to contemporary challenges, particularly ever-widening inequality and the pandemic. These constitute intersecting catastrophes that need scientific attention. Such problems bring into focus “neglected negatives” that may be fueling current difficulties, including greed, indifference, and stupidity. Anger, which defies easy characterization as positive or negative, also warrants greater scientific study. Going forward I advocate for greater study of domains that likely nurture good lives and just societies – namely, participation in the arts and encounters with nature, both currently under study. Overall, my entreaty to PP is to reckon with persistent problems from its past, while striving toward a future that is societally relevant and virtuous.

## Introduction

I have studied psychological well-being for over 30 years (Ryff, 1989, 2014, 2018), seeking to define its essential features as well as learn about factors that promote or undermine well-being and probe how it matters for health. I bring this past experience and expertise to thinking about positive psychology (PP), noting that I have never considered myself a positive psychologist, mostly because it has always seemed misguided to me to partition science by valence. Everything that interests me involves complex blends of good and bad things, what Rilke called the beauty and terror of life. With these ideas in mind, I reflect about the future of PP by first looking at its past to highlight what it has, or has not, contributed over the last two decades. My views represent personal observations from an outsider who, from the outset, was dubious about the point of launching the PP movement.

I begin with a look at early critiques, including my own, that distilled various concerns about the launching of PP. Some of those problems have endured, such as the failure to embrace the deeper history of psychology and related fields that have long addressed optimal human functioning. This distortion undermines the building of cumulative and coherent knowledge, while also contributes to insularity within PP. Additional past critiques, some from within PP, emphasized the need to put negative and positive experience together, as in dialectical approaches. I made similar points along the way. For this essay, I describe work outside the PP umbrella doing exactly that, drawing largely on the Midlife in the United States (MIDUS) national longitudinal study^1^, which I have led over the past two decades.

Returning to PP, on the topic of scientific progress I highlight select contributions over the past 20 years, but again underscore that most of these topics predated PP. On the downside, two notable problems are discussed: (1) poorly constructed measures of well-being and problematic findings, which contradict the claim that PP rests on solid science; and (2) widespread use of homogeneous research samples (white, well-educated, Western) in PP, thereby ignoring how race/ethnicity, socioeconomic status, and culture matter for positive human functioning. Linked to these problems is widespread pursuit of financial profit, purportedly grounded in rigorous scientific findings. Such commercialization, illustrated by products and shopping carts on websites, makes clear that PP has become a major business. Money-making, I observe, is a strange counterpoint to the recurring emphasis on character strengths and virtue. Financial gain raises additional issues of ethical oversight and quality control in what is being sold.

Going forward, PP and the human sciences in general need to address contemporary societal problems. I focus on ever-widening inequality, now compounded by the pandemic. What we know is that the suffering is not occurring equally, but is happening disproportionately among those who were already vulnerable. These difficulties bring into high relief topics that psychology has largely neglected. Among pernicious negatives of our era that may be fueling the problems we see are greed and indifference, especially among the privileged, as well as stupidity, which seems to cut across educational strata. Anger is another important contemporary emotion that defies easy characterization as positive or negative. These topics stand in marked contrast to what PP was meant to correct – namely, the preoccupation with psychopathology, weakness and damage (Seligman and Csikszentmihalyi, 2000).

Looking ahead, I examine factors that may be key in nourishing good lives and just societies, such as active engagement with the arts, broadly defined. Widespread initiatives are moving in this direction, though few emphasize the critical role of the arts in understanding human suffering, which I bring into high relief. A key question is whether great literature, music, poetry, painting, and film can activate caring and compassion, particularly among the advantaged. Encounters with nature constitute another domain for nourishing good lives, while also strengthening commitments to take care for our planet. I note currently unfolding work along these lines.

## Looking Back

### Early Critiques of Positive Psychology

Most cite Seligman and Csikszentmihalyi (2000) as the definitive statement of what PP was about and why it was needed. The essay began with the authors describing what led each of them to believe that psychology as a discipline was preoccupied with “pathology, weakness, and damage” (p. 7). These assertions were remarkably at odds with extensive literatures on the positive in clinical, developmental, existential, and humanistic psychology – decades of prior work, much of which I drew on to formulate an integrative model of psychological well-being (Ryff, 1989). Instead, most of the foundational exegesis was devoted to describing the 15 articles that followed. All represented longstanding programs of research on such topics as evolution, subjective well-being, optimism, self-determination, maturity, health, wisdom, creativity, and giftedness. These realms were themselves notably at odds with the assertion that psychology was preoccupied with the negative, a point strangely missed by the founders of PP.

Three years later *Psychological Inquiry* published a target article titled “Does the Positive Psychology Movement Have Legs?” (Lazarus, 2003), followed by numerous commentaries. Ryff (2003) found fault with many aspects of the Lazarus critique (e.g., subjectivism, dimensional versus discrete models of emotion, and cross-sectional research), most of which I clarified were not problems specific to PP. On the topic of emotion, however, I argued for joint focus on negative and positive emotions because “…bad things happen to people, and the healthy response is to feel the sadness, pain, frustration, fear, disappointment, anger, or shame resulting from the adverse experience. However, good things also happen to people, and the healthy response is to feel joy, pride, love, affection, pleasure, or contentment from such experience positive experiences. Thus, the capacity for experiencing and expressing both realms of emotion is central to healthy functioning.” (p. 154).

The unsatisfactory Lazarus critique meant that the central strengths and limitations of PP had not been addressed. On the credit side of the ledger, I praised the special issue for bringing together in the same forum research programs that addressed positive, healthy, adaptive features of human functioning, but underscored that everything assembled came from longstanding programs of prior research. *Nothing meant to exemplify this new movement was new*: “This myopia about past and present is damaging not for the superficial reason of taking credit for advances already contributed by others but for more serious problems of increasing the likelihood of reinventing wheels, both conceptual and empirical, such that science fails to be incremental and cumulative” (Ryff, 2003, p. 155).

To illustrate historical precursors, I drew on Coan’s (1977) *Hero, Artist, Sage, or Saint.* It described centuries of scholarly efforts to depict the more noble attributes of humankind, such as the ancient Greeks’ emphasis on reason and rationality, St. Augustine’s emphasis on close contact with the divine, the Renaissance emphasis on creative self-expression, and the poets and philosophers of the Enlightenment. I also noted James (1902/1958) eloquent writings about healthy-mindedness juxtaposed with the sick soul, along with others who formulated individuation (Jung, 1933; Von Franz, 1964), ego development (Erikson, 1959), maturity (Allport, 1961), self-actualization (Maslow, 1968), the fully functioning person (Rogers, 1961), and positive mental health (Jahoda, 1958).

My own work on well-being (Ryff, 1989) had drawn extensively on these sources, while Ryan and Deci’s (2001) review of hedonic and eudaimonic well-being distilled other philosophical precursors. I noted other contributions on positive topics, such as studies of ego development (Loevinger, 1976), adult personality development (Helson and Srivastava, 2001), generativity (McAdams and St. Aubin, 1998), the human quest for meaning (Wong and Fry, 1998), effective coping and self-regulation (Carver and Scheier, 1998), and proliferating research on human resilience and post-traumatic growth (Tedeschi et al., 1998; Luthar et al., 2000). My point: “Taken as a whole, this impressive array of current and past research on the upside of human condition leaves one wondering what all the fanfare has been about. Positive psychology is alive and well, and it most assuredly has legs, which stretch back into the distant history of the discipline. *It is only from particular vantage points, such as clinical or abnormal psychology that the positive focus constitutes a novelty.* For other subfields, especially life-span developmental and personality psychology, there has always been a concern for healthy, optimal human functioning. Perhaps the main message in the positive psychology initiative is thus how deeply entrenched and divided are the subfields within which psychologists work” (Ryff, 2003, p. 157). Unfortunately, this failure to consider relevant wider literatures has persisted through time. More than a decade later, the positive in PP was defined entirely from “Three Foundational Documents” (Pawelski, 2016), which included Seligman (1999) and Seligman and Csikszentmihalyi (2000), and an unpublished paper from a 2000 conference in Akumal, Mexico organized by Seligman. Effectively, all meanings of the positive in PP emanated from its founder, thus more deeply entrenching the historical myopia.

My 2003 essay concluded with a call for psychology to organize its house of strengths and to be circumspect about generating new assessments: “Those who would add to the many tools already available need to be clear that they are not contributing to clutter – that is, generating instruments that are redundant with extant measures.” (p. 157). The concern went unheeded, as I detail later.

### Calls to Put Negative and Positive Realms Together

Wong (2011) advocated for a balanced and interactive model of the good life: “the development of character strengths and resilience may benefit from prior experience of having overcome negative conditions” (p. 70). The call to maximize positive affect and minimize negative affect could also create a “happy person as a well-defended fortress, invulnerable to the vicissitudes of life” (King, 2001, p. 53). New to the discourse, Wong called for a balance between individualist and collectivist orientations, thereby signaling the need to address cultural issues. Similarly, Lomas and Ivtzan (2016) called for second wave positive psychology to recognize the insufficiency of the admonition of first wave PP to go beyond a psychology preoccupied with disorder and dysfunction. Negative states could be conducive to flourishing, calling again for recognition of the dialectical nature of wellbeing. Five dichotomies were examined: optimism versus pessimism, self-esteem versus humility, freedom versus restriction, forgiveness versus anger, and happiness versus sadness. Within each, the value of both sides was described. These ideas aligned with other prior work, such as Carver and Scheier’s (2003) observation that doubt and disengagement play critical roles alongside commitment and confidence as well as Larsen et al. (2003) emphasis on co-activation of positive and negative emotions that allow individuals to make sense of stressors and gain mastery over them.

At the 6th European Conference on PP in Moscow, I spoke about “Contradiction at the Core of the Positive Psychology Movement: The Essential Role of the Negative in Adaptive Human Functioning” (Ryff, 2012), beginning with a quote from Dostoyevsky’s *Notes From the Underground:* “And why are you so firmly and triumphantly certain that only what is normal and positive – in short, only well-being is good for man? Is reason mistaken about what is good? After all perhaps prosperity isn’t the only thing that pleases mankind. Perhaps he is just as attracted to suffering. Perhaps suffering is just as good to him as prosperity.” I then drew on Mill’s (1893/1989) *Autobiography:* “Those only are happy, I thought, who have their minds fixed on some object other than their own happiness, on the happiness of others, on the improvement of mankind, even on some art or pursuit, followed not as a means, but as itself an ideal end. Aiming thus as something else, they find happiness by the way.”

Arguing that psychology should not be partitioned by valence because all lives encompass both positives and negatives, I provided three examples of how they might come together. In the first, the positive is construed as an *antidote* to the negative, such as how positive emotions can help undo negative emotions (Fredrickson, 1998), or how psychological well-being can help prevent relapse of depression or anxiety (Fava et al., 1998; Ruini and Fava, 2009). In the second, the negative is seen as the *route or path* to the positive, as in trauma contributing to personal growth (Tedeschi et al., 1998), or the expression of negative emotion fostering relational intimacy (Reis, 2001), or the expression of negative emotion in childhood contributing, via skilled parenting, to emotional development (Gottman, 2001). In the third, the positive and negative emotions are inextricably linked, such that embedded within every negative is a positive and within every positive is a negative. This dialectical perspective is more common in interdependent cultural contexts, with our findings (Miyamoto and Ryff, 2011) showing that Japanese adults report experiencing both positive and negative affect, whereas United States adults report mostly positive affect. The dialectical emotional style was also linked with better health (fewer physical symptoms) in Japan compared to the United States.

Around the same time, McNulty and Fincham (2012) issued an important new challenge to PP: to consider that psychological traits and processes are not inherently positive or negative, but can be either depending on the context in which they occur. This insight was illustrated with interpersonal research (longitudinal studies of marital partners). Four putatively positive processes (forgiveness, optimism, benevolent attributions, and kindness) were shown to be beneficial, or harmful, depending on the context in which they occurred. For example, whether forgiveness was linked with self-respect differed by levels of agreeableness of one’s partner. Martial satisfaction over time also varied depending on whether attributions for spouses’ undesirable behaviors were more or less benevolent. This work, including numerous other examples, offered compelling evidence that simplistic characterizations of phenomena as positive or negative are misguided.

### Integrative Work Outside the Positive Psychology Umbrella

Extensive research not part of PP has brought negative and positive aspects of human experience together. To illustrate, I describe select findings from the MIDUS (Midlife in the United States) national longitudinal study (see text Footnote 1), which is based on diverse probability samples, thereby facilitating analyses of how well-being and health vary by age, race, gender, and socioeconomic status. A counterpart study in Japan (MIDJA) has illuminated cultural differences in well-being and health. MIDUS has unprecedented depth in high quality measures of hedonic well-being (life satisfaction, positive, and negative affect), eudaimonic well-being (autonomy, environmental mastery, personal growth, positive relations with others, purpose in life, and self-acceptance), optimism, sense of control, personality traits, generativity, social responsibility, and social ties with spouse/significant other as well as parents during childhood. Deeply multidisciplinary in scope, MIDUS has facilitated linkage of all of the above variables to epidemiology, biology, neuroscience, and genetics. Most importantly, MIDUS data are publicly available and are widely used by scientists around the world.

Many findings have combined positive and negatives. For example, Morozink et al. (2010) showed that those with lower educational attainment had elevated levels of IL-6 (interleukin-6, an inflammatory marker implicated in numerous diseases) but higher psychological well-being buffered against such effects. Miller et al. (2011) showed that those from lower socioeconomic backgrounds had increased risk for metabolic syndrome in adulthood, but maternal nurturance buffered such risk. Resilience findings (see Ryff et al., 2012) showing that positive psychosocial factors afforded protection against poor health and physiological dysregulation in the face of various challenges (aging, inequality, early life adversity, cancer, loss of spouse). Breaking new ground, multiple studies have documented that higher purpose in life predicts increased length of life and better health behaviors (Ryff and Kim, 2020). Regarding underlying mechanisms, Heller et al. (2013) showed that sustained activation of reward circuitry in the brain predicted higher eudaimonic well-being as well as better diurnal regulation of cortisol. Personality researchers have studied “healthy neuroticism,” with findings from multiple international studies showing that neuroticism is less strongly linked with poor health behaviors (smoking, inactivity) among those who are high in conscientiousness (Graham et al., 2020).

With regard to race, MIDUS has advanced knowledge of the Black-White paradox in health (Keyes, 2009) – i.e., despite inequality and discrimination, Blacks show higher levels of flourishing and lower levels of mental disorders than Whites. Keyes (2005, 2007) also revealed neglected types of mental health in the general population by jointly examining mental distress (depression and anxiety) and well-being (emotion, psychological, and social). In contrast to those who are flourishing (high well-being and no mental distress) are those who are languishing, defined as not suffering from mental distress but having low well-being. Declining well-being over time also predicted increased subsequent risk of mental distress (Keyes et al., 2010), while positive mental health predicted subsequent recovery from mental illness (Iasiello et al., 2019). Space does not permit the details, but many findings from MIDUS and MIDJA have documented cultural differences in how emotion and well-being matter for health and biological risk (Miyamoto and Ryff, 2021).

To reiterate, I include the above glimpse at MIDUS research is to underscore the need for greater interplay and exchange between the field of PP and much parallel science being done by those who do not view themselves as positive psychologists and are not publishing in journals aligned with PP or happiness.

## Recent Work in Positive Psychology

This section first below examines select areas of research that represent forward progress of PP over the past two decades. Then I note recent evaluative overviews of PP from those within the field. Some of their concerns are elaborated in the next sections on what I see as problems within PP science: first, the promulgation of poor instruments for assessing well-being, and second, the reliance on largely privileged, homogeneous samples for conducting PP research.

### Forward Empirical Progress

Whether the science of PP is moving constructively forward can examined in various ways. Rather than conduct a systematic review of empirical findings, I choose to focus on chapter-writing, mostly from 3rd Edition of the *Oxford Handbook of Positive Psychology* (Snyder et al., 2021). Unlike journal articles, chapters allow authors to combine many advances on particular topics over time thereby offering a narrative overview of multiple findings. The book includes 68 chapters written by 153 authors, 86% of whom were from the United States.

In the study of emotion, multiple lines of progress are evident. The broaden and build theory continues to evolve by showing short- and long-term benefits of positive emotions in multiple domains, including thoughts, actions, stress, health, physiological and neurological connections (Tugade et al., 2021). Studies of positive affectivity, a trait composed of different components (joviality, self-assurance, and attentiveness) have also progressed via linkages to other constructs (extraversion, happiness, and well-being) as well as psychological disorders, health, marital and job satisfaction, and cultural issues (Naragon-Gainey and Watson, 2021). Positive affect has been linked to longer life, lower incidence of disease, better recovery from disease and better overall health (Hunter et al., 2021), with calls for further work on mechanisms, culture, and technology. The emotional approach to coping (EAC) shows evidence on the intentional use of emotional processing and expression to manage adverse circumstances, such as infertility, sexual assault, diabetes, cancer (Moreno et al., 2021), while calling for more work on interventions, including who benefits (which contexts).

Happiness studies have examined ways in which happy and unhappy people respond to social comparisons, make decisions, and reflect (Boehm et al., 2021), along with strategies (experiments and activities) to improve happiness and formulation of underlying mechanisms. Veenhoven (2021) reviewed differences in happiness across nations and linked them to important questions about what governments can or cannot do to raise levels of happiness, thus reaching toward issues of public policy. A unified model of meaning in life was advanced, underscoring the need for conceptual integration in this growing area of science (Steger, 2021). Positive aging was covered via multiple positive formulations that have been extensively studied, in some cases with interventions (Nakamura and Chan, 2021).

Shifting to life outlooks, how the future is construed was covered with work on optimism showing that those who expect good things to occur have higher well-being, better health, and higher quality social ties, partly attributable to how they cope with adversity (Mens et al., 2021). Detrimental consequences of hope were considered, while calling for greater work on the origins of hope and cultural issues. Hope, defined as the perceived ability to achieve desired goals via pathways and agency, was examined with a goal pursuit process model and linked to academic and athletic performance, health and well-being, social relationships, and work (Rand and Touza, 2021). Resilience, the capacity for positive adaptation in the face of significant adversity, was examined in models that illuminated self-regulation skills, good parenting, community resources and effective schools (Cutuli et al., 2021). Strategies for reducing risk, building strengths and mobilizing adaptive systems were future directions.

Positive mental health was covered with a thoughtful historical perspective and overview of current conceptualizations and measures (Delle Fava and Negri, 2021), examined from life course and cultural perspectives. Illustrating methodological novelty, Tarragona (2021) considered the benefits of personal narratives and expressive writing on mental health and physical health (immune function and cardiovascular health), particularly in the context of trauma. Dominant approaches to mental health interventions (psychotherapy, counseling, and coaching) were examined for commonalities and differences in time perspectives, therapeutic strategies and recipients (Ruini and Marques, 2021), while emphasizing the need for professional regulation and oversight.

Several chapters covered interpersonal themes. Attachment theory was presented as a framework for studying positive relationships (Mikulincer and Shaver, 2021) via links between mental representations of attachment security and how they matter for diverse outcomes (health, social adjustment or interpersonal conflict, and personal growth). Relationship complexities were examined, underscoring both meaningful rewards and substantial risks of close social ties (Gable and Maisel, 2021). They highlighted positive processes, involving positive emotions, intimacy, growth of self-concept, and benefits of sharing positive events. Past research on empathy was reviewed and emerging work on the neuroscience of empathy described (Duan and Sager, 2021). How empathy relates to racial/ethnic diversity, multiculturalism, and social justice were future directions. Forgiveness was described in terms of the methods used and the differentiation of various antecedents, some intrapersonal (empathy, personality, attributions, and religion) and others interpersonal (closeness and conciliatory behavior) (Tsang and Martin, 2021). Whether forgiveness is uniformly positive was considered.

Pawelski and Tay’s (2021) described efforts to connect PP to the humanities through new conceptual analyses and various interventions. Silvia and Kashdan (2021) examined curiosity and interest, framed as recognizing, seeking out, and preferring things outside one’s normal experience. How these tendencies matter for well-being is under study in the laboratory and everyday life. Courage, defined as facing personal risks in pursuit of worthy goals, was examined historically and via modern theory and measurement tools focused on volition, goals, and risk (Pury et al., 2021). Humility, formulated as accurate and modest self-presentation and being other-oriented, showed steady progress in empirical findings from 2000 to 2015 (Worthington et al., 2021).

In sum, considerable evidence reveals forward progress on important topics in PP. Even though most areas of inquiry predated PP, it is useful to bring such contributions together to convey the range and diversity of topics on adaptive human functioning. At the same time, several chapters in the collection were not current in coverage, and some had a paucity of empirical findings. All ended with future questions. An interesting question is whether these have evolved over the past 20 years, or are largely similar to where the field was back then. Before addressing problematic areas of PP science, I next examine evaluative reviews from within the PP field.

### Overarching Concerns About Positive Psychology

Lomas et al. (2021) call for PP to broaden toward complexity – go beyond the individual toward analysis of groups, organizations, and broader systems as well as to embrace diverse methodologies. Better understanding of context (historical, social, cultural, and institutional) was also emphasized. Contextual approaches were illustrated with positive organizational scholarship (Cameron et al., 2003), positive educational approaches in schools (Waters et al., 2010), and family-centered positive psychology (Sheridan et al., 2004; Henry et al., 2015). Lomas et al. (2021) called for greater ethical oversight of the ever-expanding cadre of PP practitioners from applied programs: “…unless practitioners are affiliated to a particular profession, they may be operating outside the advice and provisions of any set of ethical guidelines” (p. 16).

Kern et al. (2020) contrasted the rapid growth of PP with concern about exaggerated claims, inflated expectations, disillusionment, and possibly, unintentional harms. Issues of over-promising and under-delivering in programs with individuals, schools, the workplace, and communities were noted. To help the field mature, they advocated for systems informed PP, which would clarify epistemological, political, and ethical assumptions and commitments. The implications of such ideas for research and practice were examined.

van Zyl (2022) reviewed criticisms and concerns about PP, including the lack of a unifying metatheory that underpins the science as well as fundamental ideas for how positive psychological phenomena should be researched. Related criticisms were that PP has borrowed most of its theories from social, behavioral and cognitive psychology, thereby advancing few of its own unique perspectives. There is the problem of terminological confusion – e.g., using terms like flourishing or well-being interchangeably when operationalizations of them are notably different, or failing to recognize the possible overlap among putatively distinct topics, such as grit, conscientiousness, or diligence. Inconsistency in the factorial structures of various measurement models is a further problem. The fact that most PP has failed to produce significant or sustainable changes was noted, along with its cultural (Western) biases.

Taken together, I agree with most of the above assessments and further illustrate them below.

### Problems in Positive Psychology Science: Flawed Conceptualization and Measurement of Well-Being

I bring my expertise in the study of psychological well-being to how some have approached this topic in PP. As noted above, I foresaw problems of measurement clutter at the dawning of PP (Ryff, 2003). My prediction was prescient and needs attention, given growing interest in the measurement of well-being across scientific disciplines. A recent edited volume (Lee et al., 2021) included scrutiny of multiple measurement approaches along with an animated exchange among contributors (Ryff et al., 2021a,b; VanderWeele et al., 2021a,b) on the pluses and minuses of various assessment strategies. What came into high relief was concern about the proliferation of thin, poorly validated measures that are undermining quality science in the study of well-being.

Although not considered in the above volume, Seligman and his collaborators have contributed to this problem. I offer two examples of the promulgation of poorly constructed and poorly validated measures of well-being that are at odds with claims that PP rests on rigorous science. A first study (Seligman et al., 2005) sought to validate five different interventions (gratitude visit, three good things, you at your best, using signature strengths in a new way, and identifying signature strengths). Internet-based samples were recruited through the authentic happiness website^2^; most participants were white and highly educated.

All completed baseline assessments and five follow-up assessments over a 6-month period after completion of the intervention assignment. As a general observation, the findings were overstated – most comparisons between the control group and intervention groups were not significantly different across time, nor was there coherence in when such effects were evident. There was also insufficient attention given to pre–post comparisons, which are central for demonstrating intervention effectiveness. My primary focus, however, is on the outcomes assessed – specifically, the measure of happiness.

Described as “scientifically unwieldy” (p. 413) happiness was “dissolved” into three distinct components: “(a) positive emotion and pleasure (the pleasant life), (b) engagement (the engaged life), and (c) meaning (the meaningful life).” I note the redundancy in defining each component. The source for this tripartite formulation was Seligman’s (2002) trade book, *Authentic Happiness*, which was operationalized with the Steen Happiness Index (SHI), an unpublished 20-item inventory. No evidence was provided that the inventory measures three distinct components of well-being, nor is it likely such evidence could be assembled. Many items lack face validity – i.e., they pertained to other constructs, such as optimism, positive self-regard, frustration, energy, social connection, making good choices. Adding to the befuddlement was this statement: “We continue to use the word happiness, but only in the atheoretical sense of labeling the overall aim of the positive psychology endeavor and referring jointly to positive emotion, engagement, and meaning” (p. 413). All analyses focused the atheoretical construct of happiness – i.e., the component parts were nowhere to be seen.

Next came PERMA, defined by Seligman (2011) in *Flourish*, another popular book. Added to the prior components of positive emotion, engagement, and meaning, were now two additional components: relationships and accomplishment. Again, none were explicitly defined, nor was the pronouncement about what happiness entails theoretically grounded in *anything*, nor was it linked with the extensive prior empirical literatures on subjective and psychological well-being as well as research on positive emotions (exemplified by the diverse MIDUS measures). Such obliviousness to what the field had been investigating for decades made inevitable that there would be redundancy with already validated approaches and assessment tools. Such duplication became a certainty given how PERMA was operationalized – namely, *by taking items from prior instruments* (Butler, 2011). These were transformed into the PERMA-Profiler (Butler and Kern, 2016) via multiple studies (none clearly defined) involving a large samples recruited mostly through online systems; most participants were well-educated.

Missing from the reported analyses were key preliminaries required to develop quality assessments. For example, of central importance was whether the item pools for the five components were empirically distinct (i.e., did each item correlate more highly with its own scale than another scale?). In subsequent tests of convergent validity with other measures, a further problem, not addressed, was the degree of item-overlap (redundancy), given that all PERMA items came from prior instruments. Additional analyses correlated PERMA scales with 20+ measures. For many (e.g., organizational practices, political orientation, work performance, social capital, burnout, values, self-efficacy, perceived stress, and gratitude), the relevance of these analyses was unclear.

Subsequent work showed that PERMA and subjective well-being are indistinguishable (Goodman et al., 2018). Seligman (2018) responded by calling for the need to “transcend psychometrics,” accompanied by an exegesis on the psychometrics of baseball pitching. Also offered was the observation that “SWB probably is the useful final common path of the elements of well-being” (p. 1) – presumably an effort to deflect evidence away from the clear empirical redundancy of PERMA with subjective well-being. Most incoherent was the following: “All of this is to say that a good theory of the elements of well-being helps to build well-being and that the psychometric findings that the elements correlate perfectly with overall well-being and that the elements correlate very well with each other is not very instructive when it comes to building well-being” (p. 2).

Other findings have shown questionable support for the putative five-factor structure of PERMA (Watanabe et al., 2018; Ryan et al., 2019; Umucu et al., 2020). Data from German speaking countries Wammerl et al. (2019) supported for the five-factor model but also bifactor models (Reise, 2012). My observation is that these latter methodological studies examining various multivariate structures are largely disconnected from substantive issues of what well-being is, or critical questions needed to advance the field. Those are not about dimensional structures of recycled items, but about the antecedents and consequents well-being, whether well-being is protective in the face of adversity, and whether interventions can promote well-being. On all of these questions, the above two efforts to articulate a meaningful, conceptually grounded theory of happiness that works empirically (i.e., the data support the claimed multifactorial structure) AND that is distinct from what was already in the field, have failed.

### Problems in Positive Psychology Science: Samples and Contexts

A second major problem in PP research, already illustrated in preceding sections, is the overwhelming reliance on *homogeneous, privileged samples*. This lack of diversity pervades subfields of psychology that have tended to conduct their research with readily available college students or community volunteers. Others call this the WEIRD phenomenon (Henrich et al., 2010) – doing research with western, educated, industrialized, rich, and democratic societies. Minorities and socioeconomically disadvantaged individuals are missing in such inquiries, although population research makes clear that well-being and health are linked with sociodemographic factors (Ryff et al., 2021c). Our review, which included findings from MIDUS and other large studies, made clear that numerous aspects of well-being (hedonic and eudaimonic) do, in fact, differ by age, socioeconomic status, race, and gender. These differences also predict diverse health outcomes, assessed in terms of symptoms, chronic conditions, biological risk factors, and mortality. Thanks to the MIDJA (Midlife in Japan) study, we have illuminated cultural differences in many of these same topics (Miyamoto and Ryff, 2021).

Closer to PP, I note that *Frontiers in Psychology* issued a recent call for papers to address with PPI (positive psychology interventions) work in non-WEIRD contexts (van Zyl et al., 2021). Their bibliographic analyses showed that only about 2% of PPIs to date have been conducted with vulnerable groups, or in multi-cultural contexts. Clearly, a major need going forward is the importance of reducing the bias toward Western (often United States) samples of privileged people whose lives are clearly not representative of those from other cultural contexts as well as focusing on disadvantaged groups within such contexts.

### The Commercialization of Positive Psychology: Needed Oversight

It is without question that PP has become a big business (Horowitz, 2018). Happiness promotion involves billions of dollars spent on popular books, workshops, counseling/coaching, apps, websites, and social media platforms. PP has entered the corporate world through happiness consulting companies that claim to “bridge the gap between cutting-edge research in the field of positive psychology and best practices within corporate and community cultures around the globe” (p. 244). Horowitz wryly observes that few promoting happiness as the route to success consider the alternative – i.e., that success leads to happiness. There is also a marked failure to address the needs of lower echelon workers, such as better wages and benefits. Instead, motivational speakers cheer on executives, managers and workers with messages consonant with positive psychology and neoliberalism. Via apps and other gadgets happiness has become a “measurable, visible, improvable entity” (p. 246), thus replacing global commitments to combat stress, misery, and illness was with relaxation, happiness, and wellness.

I will not detail the dizzying array of websites promoting happiness, flourishing, and positive psychology; they are easily found online. Instead, I ask whether the for-profit cart has gotten seriously ahead of the scientific horse. This is a matter the scientific community cannot afford to ignore because it addresses whether the evidential basis behind the proliferation of products is truly there, or has been glossed over in the frenzy to sell. Prior to the commercialization of PP, scientists had shared understanding of what is required to demonstrate intervention effectiveness, as in randomized clinical trials, a staple of the National Institutes of Health. These guidelines exist to protect the public from products that are not credible. That the advertised promise of happiness promotion may be overstated is intimated by the “Earnings Disclaimer and Statement of Individual Responsibility” from the Flourishing Center^3^. It states that “the Flourishing Center, Inc. makes no guarantees that you will achieve results similar to ours or anyone else’s.” Additional text in this format follows: YOU FULLY AGREE AND UNDERSTAND THAT YOU AND YOU ALONE ARE RESPONSIBLE FOR YOUR SUCCESS OR FAILURE. NO REFUNDS ARE AVAILABLE UNLESS STATED OTHERWISE ON A PROGRAM’S SALES PAGE.

Closer to the heart of PP, we need to ask what it means when character strengths are being sold, when virtue has become a commodity, and when PP scientists have shopping carts on their websites. There is also the matter of pricing. Horowitz (2018) describes some who are receiving $25,000 speaker fees – are these defensible in academia? Many believe we have a responsibility to share our knowledge and expertise, but not to do so in pursuit of personal profit. Scrutiny also is required regarding the content of educational programs. Here I focus on the flagship program that is presumably leading the field – namely, the Master’s in Applied Positive Psychology (MAPP) at the University of Pennsylvania, described with no shortage of hubris, as Medici II (Seligman, 2019). MAPP offers two semesters (nine courses) and a summer capstone project for a price of over $70,000. The curriculum is thinly described on the website, but if students are being taught that the theory, history, and meanings of PP (Introduction to Positive Psychology) began with Seligman and Csikszentmihalyi (2000) and other foundational documents (Pawelski, 2016), they are not getting what they paid for. Further, if PERMA is being taught as a credible tool for measuring well-being (Research Methods and Evaluation), they are being miseducated. The theoretical, empirical, and experiential nature of positive interventions (Foundations of Positive Interventions) are not detailed on the website, but if Seligman et al. (2005), reviewed above, is presented as credible evidence that PP interventions work, they are being misled.

Amidst these questions, it is important to underscore that high quality teaching materials for such programs do exist, such as the recent book on *Positive Psychology Through the Life Span: An Existential Perspective* (Worth, 2022) and another on *Positive Psychology in the Clinical Domains* (Ruini, 2017). Both offer thoughtful, *historically comprehensive* perspectives in their respective domains, which are essential features of quality education in PP.

The larger issue is the *quality* of what PP is marketing, not just in master’s programs, but also certificate programs and short-term seminars. Horowitz (2018) notes those who have expressed concerns about ethical oversight, calling for standardized nomenclature, formal training and certification guidelines, given uneven credentialing among those doing this work. Central concerns are whether the teaching in some programs is superficial and short-term practices lack scientific evidence of effectiveness. Stated otherwise, the commercialized end of PP appears to be fundamentally unregulated. “Despite all the research carried out in the field, what remains too often neglected are the who, why, and with what results ordinary consumers gain from all the money and time they spend on pursuing positive psychology by reading books, attending workshops, and carrying out recommended exercises.” (Horowitz, p. 274).

## Looking Forward: Suggested New Directions for Positive Psychology

### Societal Ills as Research Imperatives

Two major challenges of our era, ever-widening inequality and the world-wide pandemic, need scientific attention. Together, they constitute *intersecting catastrophes* (Ryff, forthcoming). Among those who were already disadvantaged, the pandemic has aggravated difficulties many were already facing plus added new challenges (unemployment, loss of healthcare, evictions due to unpaid rent, and food lines/hunger). MIDUS has been a prominent forum for investigating health inequalities, given its rich psychosocial, behavioral, and biological assessments (Kirsch et al., 2019). Our findings have linked lower education and incomes to compromised well-being, greater psychological distress, poorer health behavior, higher stress exposures, elevated biological risk factors, greater morbidity and earlier mortality (see Text Footnote 1). A unique feature of the study has been recruitment of two national samples situated on either side of the Great Recession. Over the period covered by these two samples, educational attainment in the United States improved.

Despite such educational gains, the post-Recession refresher sample reported less household income (after adjusting for inflation), lower financial stability, worse health (multiple indicators) and lower well-being (multiple indicators) than the pre-Recession baseline sample. Further work compared the two samples on measures of negative and positive emotions, showing more compromised mental health in the later refresher sample, particularly among those with lower socioeconomic standing (measured with a composite of education, occupation, income, and wealth) (Goldman et al., 2018). This worsening of mental health among disadvantaged Americans has occurred in the context of the opioid epidemic, growing alcoholism and increased death rates, including suicide, among middle-aged white persons of low SES standing (Case and Deaton, 2015; Kolodny et al., 2015; Grant et al., 2017; Schuchat et al., 2017), a phenomenon known as *deaths of despair* (Case and Deaton, 2020).

Positive psychologists need to engage with these societal changes. I note promising work already underway (Waters et al., 2021). Although human strengths constitute important protective resources in the face of adversity, it is also the case that significant challenge can sometimes disable pre-existing strengths (Shanahan et al., 2014). We found evidence of such disablement among those exposed to high levels of hardship in the Great Recession (Kirsch and Ryff, 2016). Going forward, it is critical that studies of psychological strengths in the face of pandemic stress include assessment of key sociodemographic variables such as socioeconomic status in national samples. Vazquez et al. (2020) illustrated such work in a representative sample of Spanish adults. It is critical that future PP contributions to understanding impacts of the pandemic not perpetuate the longstanding prior focus on privileged, homogeneous samples.

### Neglected Negatives Behind the Current Societal Problems

The founders of PP advocated that psychology should encompass more than psychopathology (depression and anxiety) and other forms of dysfunction. Hence, the call to elevate positive aspects of human functioning. I observe that psychology as a discipline has neglected something else: namely, a category of negative characteristics that may be implicated in the societal problems we now face. These include greed, indifference, and stupidity (Ryff, 2017, 2021a), along with anger, which is not inherently positive or negative. I cover these topics below because they reveal a possibly pernicious blind spot in the larger vision of PP: namely, that the well-being and positive human functioning of some (especially those who are disadvantaged) may be compromised by the priorities and actions of others (especially those who are advantaged). To the extent that PP ministers primarily to the better educated and economically comfortable in conveying how to get the most out of life and achieve personal potential, PP may, itself, be part of the problem.

To illustrate, I note the widespread marketing of mindfulness meditation, including to CEOs as described by Horowitz (2018) in *Happier?* Purser (2019) offers more, observing that “mindfulness programs do not ask executives to examine how their managerial decisions and corporate policies have institutionalized greed, ill will, and delusion. Instead, the practice is being sold to executives as a way to de-stress, improve productivity and focus, and bounce back from working 80-h weeks. They may well be ‘meditating,’ but it works like taking an aspirin for a headache. Once the pain goes away, it is business as usual. Even if individuals become nicer people, the corporate agenda of maximizing profits does not change.”

#### Greed

Following from the above quote, we must consider that among the malevolent forces contributing to ever-widening inequality are behaviors of excessive self-interest orchestrated by those in positions of power. These problems are empirically evident when corporate profits soar, but worker paychecks lag (Cohen, 2018), a problem described by economists as “monopsony power” – the ability of employers to suppress wages below the efficient or perfectly competitive level of compensation (Kruger and Posner, 2018). Human history shows longstanding concern about problems of greed. The ancient Greeks saw greed and injustice as violating virtues of fairness and equality, and thereby, contributing to civic strife (Balot, 2001). Dante’s *Divine Comedy* (Dante’s, 1308/2006) placed sins of greed and gluttony, along with fraud and dishonesty, in his nine circles of hell. Adam Smith’s *Wealth of Nations* (Smith’s, 1776/1981) made the case for self-interest and capitalism, but recognized the problem of greed, framed as the limitless appetites of the vain and insatiable.

Some within psychology are addressing what lies behind the worship of money and selfish wealth gratification, sometimes orchestrated through fraudulent tactics (Nikelly, 2006). Motivational psychologists have studied “the dark side of the American Dream” (Kasser and Ryan, 1993), showing that those motivated by primarily extrinsic factors (financial success) have lower well-being and adjustment compared to those motivated by less materialistic values. Social psychologists have shown that those with higher social class standing have increased sense entitlement and narcissism compared to those from lower class backgrounds; those in the upper-class are also more likely to behave unethically than those in the lower-class (Piff et al., 2012; Piff, 2013). A large study of United States students examined what lies behind the widespread acceptance of inequality (Mendelberg et al., 2017) by asking them to indicate their agreement or disagreement with the statement: “Wealthy people should pay a larger share of taxes than they do now.” The main finding was that students from affluent colleges (defined by family SES background) were more likely than those from public or less affluent colleges and universities to disagree with the statement – i.e., the most privileged were also the most strongly opposed to having the wealthy pay more taxes. In addition, such tendencies were most pronounced among those who were active in college fraternities and sororities.

The seamy underside of philanthropy, usually thought of as elites doing good in the world, is also under scrutiny (Giridharadas, 2018). The Sackler family, well-known for their philanthropy in art museums around the world, offers a singular example. They owned Purdue Pharma, which created oxycontin, the highly addictive opioid painkiller that was aggressively marketed, thereby leading to massive over-prescribing. To date, more than 500,000 have died from overdose deaths. A 2021 HBO documentary, *Crime of the Century*, revealed the widespread individual actions behind this public health tragedy – within drug companies, political operatives, and government regulators, all of whom backed the reckless distribution of this deadly, but highly profitable, drug.

Some might argue that the above examples are isolated actions of those of extreme wealth and do not represent most of the rest of us. Stewart’s (2021) recent look at the new American aristocracy suggests otherwise. With a solid evidential basis, he shows that a much larger segment of the population is involved in warping our culture – i.e., how those laser-focused on career success are relying on an underpaid servant class to fuel their forward progress, while also making personal fitness a national obsession, even as large segments of the population lose healthcare and grow sicker. The privileged also segregate themselves in exclusive neighborhoods and compete relentlessly in getting their children into elite schools, which has contributed to ever-more extreme costs of higher education. Perhaps most troubling is the ethos of merit they have created to justify their advantages. Stewart powerfully distils that these people are not just around us, they are us.

#### Indifference

On this topic I have little to say other than to quote Elie Wiesel, Nobel Prize winning author and Holocaust survivor: “I believe that a person who is indifferent to the suffering of others is complicit in the crime. And that I cannot allow, at least not for myself. The opposite of love is not hate, it’s indifference.” In the present era, such indifference to the widespread suffering of others must be studied and documented. It is a character weakness that psychologists should try to understand – where does it come from? How is it enacted? What are its consequents?

#### Stupidity

Marmion’s (2018) tongue-in-cheek edited collection on the *Psychology of Stupidity* warrants consideration, given psychology’s long preoccupation with studying intelligence (of multiple types) and cognitive capacities (also of multiple types). The book offers a taxonomy of morons and links stupidity with established topics (cognitive bias, narcissism, and negative social networks). Wisely, Marion asserts: “No matter what form it takes, stupidity splatters us all. Rumor has it that we ourselves are the source of it. I am no exception” (p. ix). The kind of stupidity that most interests me and needs critical study is the swallowing of lies, or being duped by others. Lies are perpetrated by people in high or low places, but the essential question is why they have impact – why they are believed. Some in the clinical realm have examined such questions, focusing on those who lie with impunity, sometimes revealing clear sociopathy (Peck, 1983; Stout, 2006). We need more science about these assaults on the truth and why they have become such pervasive part of contemporary life. My hypothesis is that all levels of human experience (personal ties, the workplace, communities, and societies) are damaged by the swallowing of lies, whether knowingly or unknowingly.

#### Anger

Often depicted as toxic, anger is sometimes legitimate as Aristotle understood. He reminded that at the right time, to the right degree, and for the right reasons, anger can be a powerful and needed response. Indeed, its neural underpinnings look more like positive affect than depression or anxiety (Harmon-Jones et al., 2011). Anger may be uniquely justified vis-à-vis profoundly unequal life opportunities. Mishra’s *Age of Anger* (Mishra’s, 2017), offers an astonishing integration of history, philosophy, literature, politics, economics, and cultural studies on the topic. He begins with this: “Individuals with very different pasts find themselves herded by capitalism and technology into a common present, where grossly unequal distributions of wealth and power have created humiliating new hierarchies. This proximity is rendered more claustrophobic by digital communications and the improved capacity for envious and resentful comparison” (p. 13). Drawing on Arendt, Mishra describes *existential resentments* that are poisoning civil society and fueling authoritarianism.

Most powerful is Mishra’s portrayal of the distinct philosophies of Rousseau and Voltaire, eighteenth century interpreters of life. Voltaire praised material prosperity and consumerism, boldly professing his love of conspicuous consumption. Rousseau reminded that the ancients spoke incessantly about morals and virtue whereas the French *philosophes* spoke only of business and money. He saw the new commercial society as acquiring features of class division, inequality, and callous elites whose members were corrupt, hypocritical and cruel. According to Mishra: “What makes Rousseau, and his self-described ‘history of the human heart,’ so astonishingly germane and eerily resonant is that, unlike his fellow eighteen-century writers, he described the quintessential inner experience of modernity for most people: the uprooted outsider in the commercial metropolis, aspiring for a place in it, and struggling with complex feelings of envy, fascination, revulsion, and rejection” (p. 90). Although Rousseau’s books were best sellers in his era, they are rarely invoked in current discourse. He castigated the Enlightenment *philosophes* for their self-love and self-interest, writing that *amour propre* (McLendon, 2009) was a dangerous craving to secure recognition for self over others and an insatiable ambition to raise personal fortunes. These observations need serious examination vis-à-vis the thriving business of PP – to what extent are self-interest and personal ambition the central motives behind what is being sold?

Returning to empirical science, I note that MIDUS includes multidimensional assessments of anger, from over 20 publications have been generated (see Text Footnote 1). Anger expression has been linked to multiple indicators of health (sleep, cognitive function, inflammation, and allostatic load) as well as to race/ethnicity, socioeconomic status, early life adversity, and cultural context.

### What Nurtures Our Better Selves: The Arts and Humanities

To those who find my views to be overly negative, I end this section with more hopeful topics. I note that my career journey has reflected this dual focus on the forces that both undermine as well as nurture positive psychological functioning (Ryff, 2022). As stated at the outset and multiple times long the way, I have always believed both are fundamental parts of the human experience. I begin this part with distant observations from Matthew Arnold, who in *Culture and Anarchy* (Arnold, 1867/1993), emphasized that freedom should be employed in the service of higher ideals and further noted that these ideals are critically important during times of great peril, such as pandemics and wars. For him, culture was the study of perfection tied to the moral and social passion for doing good.

I have long believed that the arts (broadly defined) and humanities (history and philosophy) can help us discern how to do good and be well (Ryff, 2019). Growing research is now linking diverse art (music, literature, poetry, art, film, and dance) to health (Fancourt, 2017; Fancourt and Finn, 2019). To maintain a thread to current societal challenges, I here consider the arts in a somewhat different way – namely, whether they might be venues for nurturing compassion and insight about human suffering, which has become so widespread. Starting with contemporary film, multiple examples (e.g., *The Florida Project, American Honey, Paterson, Parasite*, and *Nomadland*) reveal the lived experience of inequality, including descending into prostitution to feed a child, growing up with addicted parents, having dreams of self-realization stymied, experiencing homelessness, and working in physically-difficult, mind-numbing jobs. These works also portray the poetry in disadvantaged lives, including cleverness and resourcefulness vis-à-vis insensitive elites. The relevance of these domains for contemporary science, largely unstudied, is whether such inputs increase quotients of caring and compassion, and possibly challenge the complacency and indifference among those who are not suffering. Such questions elevate themes of social justice in ongoing research on well-being and health, while pointing to the arts as possible venues for informing and mobilizing individual and societal action.

The visual arts may also powerfully activate compassion vis-à-vis the pandemic or contemporary conflicts. The self-portrait of the Austrian artist, Egon Schiele, painted in 1912 and looking gravely ill before his death at age 28 from the Spanish flu, which also took his wife and their unborn child, is an example. Kandinsky painted *Troubled* in 1917, an abstract work of turbulence and trauma created during the Russian revolution when he was lived in Moscow and had a child die of malnourishment. A last visual example comes from over 1,000 watercolors painted from 1940 to 1942 and brought together in *Charlotte Salomon: Life? Or Theater?* (Salomon, 2017). Born in 1917, this woman experienced multiple suicides in her family during her brief lifetime. She was a student at the Berlin Fine Arts Academy and in 1938 fled to southern France where an intense period of creativity unfolded. Next to a series of paintings depicting multiple faces with dramatic eyes and sad countenances, she wrote: “I realized that no heaven, no sun, no star could help me if I did not contribute by my own will. And then I realized that actually I still had no idea who I was. I was a corpse. And I expected life to love me now. I waited and came to the realization: what matters is not whether life loves us, but that we love life.” This insight about loving life had tragic salience: she was transported to Auschwitz in 1943 where, at age 26 and 5 months pregnant, she died.

Literature is another powerful realm for revealing travesties of the human condition. In *A Tale of Two Cities* (Dickens, 1859/2004), Charles Dickens brought horrors of the French Revolution to the hearts and minds of his readers. We learned of the awful lives of those imprisoned within the Bastille, and after it was stormed, the executions by guillotine at the Place de La Concorde in Paris. The bloodbath of class retribution took more than 1,200 lives, including the French Queen and King. Here is how Dickens described the context: “…the frightful moral disorder born of unspeakable suffering, intolerable oppression, and heartless indifference” (p. 344). At the core of the book is Madame DeFarge, the tigress quietly knitting, observing, and overseeing the acts of vengeance. Near the end, we have insight into her fury, learning that her younger sister was the victim of shameless male aristocrats who carelessly exploited her and destroyed her life and family.

Two contemporary books of fiction address the current migration crisis. Mohsin Hamid’s *Exit West* (Hamid’s, 2017) describes the awful realities of refugees whose lives have been stolen out from under them, only to be subjected to endless trauma as they try to find another home. Another recent work, *Call Me Zebra* (Van der Vliet Oloomi, 2018), winner of the 2019 PEN/Faulkner award for fiction, tracks a family escaping from Iran by foot. The mother dies along the way, but the father and daughter eventually make their way to New York. The family is a group of anarchists, atheists, and autodidacts who took refuge in books; their distilled philosophy: “Love nothing except literature, the only magnanimous host there is in this decaying world…. The depth of our knowledge, the precision of our tongues, and our capacity for detecting lies is unparalleled” (p. 8). Memorization is key; thus, sprinkled throughout the book are quotes from Nietzsche, Omar Khayyam, Dante, Goethe, Rilke, Kafka, Cervantes, Garcia Lorca, Dali, and Picasso – “These writers’ sentences deposited me at the edge of the unknown, far from the repulsive banality of reality others refer to as life” (p. 205).

I conclude with examples of satire vis-à-vis experiences of oppression and want. Jonathan Swift’s, *A Modest Proposal*, written in Swift’s (1729), was put forth with the stated intent of preventing the children of the poor people in Ireland from being a burden to their parents or the country, as well as to make them beneficial to the wider public. Swift began by describing female beggars in Dublin followed by their many children, all in rags, importuning every passing person for alms. He elaborated on the numerical scope of the problem and then observes that these young children cannot be fruitfully employed until they are around age twelve. Swift thus suggests that these children, if well nursed for their first year, be sent to England to provide “a most delicious nourishing and wholesome food, whether stewed, roasted, baked, or boiled; and I make no doubt that it will equally serve in a fricassee, or a ragout” (p. 3). Calculations were included to show the financial benefits that would follow. This satirical hyperbole mocked the heartless attitudes toward the poor among the British as well as their policies toward the Irish in general. The book is widely recognized as one of the greatest examples of sustained irony in the history of the English language.

Moving to the present, Paul Beatty’s *The Sellout* (Beatty’s, 2015) won the Man Booker Prize and was praised as “Swiftian satire of the highest order.” The book covers race relations in the fictional township of Dickens (meaningfully named), California, a place where residents are left to fend for themselves. With masterful humor, Beatty parodies everything – from contemporary psychology to “slapstick racism” to public transportation to depict the obstacles of being poor and black in racist America. Sister cities for Dickens are identified: Chernobyl, Juárez, and Kinshasa – all known for their pollution, poverty, and dysfunction. The satire and razor-sharp wit reveal what it means to exist in a culture saturated with negative stereotypes.

To summarize, I have emphasized the role of the arts in awakening the wider public to human suffering. Central questions for science and praxis are whether these inputs can effectively increase needed supplies of compassion and empathy, while perhaps also provoke awareness of complacency among those who are comfortable, if not indifferent. Such topics can and should be studied, including in experimental and educational contexts. The National Endowment for the Humanities regularly tracks who partakes of the arts and further shows variation therein by educational status. Such practices are fundamentally not different from studying health behaviors (smoking, drinking, and exercise). These parts of living, focused on the content of what people are taking in, need to part of large epidemiological studies, where they could be linked with other important topics such as reported levels of social responsibility and caring (Ryff and Kim, 2020) as well as their views about who should be taxed at what levels (Mendelberg et al., 2017).

### What Nurtures Human Flourishing: The Natural Environment

Nature is powerfully present in the visual arts and music as well and has been throughout human history. I have recently covered these topics elsewhere, including nature’s role in nurturing the human spirit (Ryff, 2021b) and here highlight some of that work. My overall messages are that those interested in understanding influences that nurture good lives as well as a concern for our planet need to bring encounters with nature into their scientific studies, including interventions designed to promote diverse aspects of well-being and health.

Vibrant research is now investigating how nature contributes to human flourishing (Capaldi et al., 2015; Mantler and Logan, 2015). These ideas take on greater salience as more of the world’s population live in nature-impoverished urban milieus. Multiple theories have been invoked to explain how we benefit from nature, such as the biophilia hypothesis from evolutionary thinking, which suggests that our human ancestors depended on connecting with nature to survive (Kellert and Wilson, 1993), or stress-reduction theory (Ulrich et al., 1991), which proposes that past exposures to unthreatening natural environments contributed to survival via stress-reducing physiological responses. Other perspectives consider roles of the natural environment in addressing existential anxieties, such as meaning in life, isolation, freedom, and death (Yalom, 1980). Eco-existential positive psychology (Passmore and Howell, 2014) thus describe how restorative experiences with nature might contribute to sense of identity, multiple forms of happiness, meaning, social connectedness, freedom, and awareness of one’s mortality.

Empirical evidence has linked encounters with nature to high hedonic well-being, both short and long-term, and to aspects of eudaimonic well-being (Capaldi et al., 2015; Mantler and Logan, 2015; Triguero-Mas et al., 2015). Some inquiries have examined intervening mechanisms, such as increased physical activity, increased social contact, stress reduction and restoration of cognitive attention. The focus on green spaces underscores growing concerns about urbanization, loss of biodiversity, and environmental degradation. Increasingly dire consequences of climate change (droughts, wildfires, and floods) have also led to research on pro-nature behaviors that support conservation of nature and biodiversity. Richardson et al. (2020) conducted an innovative population survey in the United Kingdom examining links between pro-nature actions with time spent in nature as well as knowledge of and concerns about nature.

Nature as a source of inspiration and uplift is pervasively present in poetry, literature, music, art, history, and philosophy. An example is the life of Alexander von Humboldt (1769-1859), beautifully written about in *The Invention of Nature* (Wulf, 2016). Primarily a scientist, naturalist, and explorer (of South America and Siberia), Humboldt influenced many of the great thinkers of his day, including Jefferson, Darwin, Wordsworth, Coleridge, Thoreau, and Goethe. Humboldt was ahead of his time in thinking about the degradation and exploitation of nature, warning that humankind had the power to destroy the natural environment, the consequences of which would be catastrophic. He wanted to excite a ‘love of nature’ and thereby, revolutionized how the natural world was seen. He believed that nature speaks to humanity in a voice “familiar to our soul” (p. 61), thereby aligning himself with the Romantic poets of his time who believed nature could only be understood by turning inward.

The educator Mark Edmundson uses great literature and poetry to nurture well-being, including the ideals needed by the human soul such as courage, contemplation, and compassion (Edmundson, 2015). In *Why Read* (Edmundson, 2004). Edmundson elaborates what a liberal, humanistic education can contribute to personal becoming. Apropos of Humboldt and his contemporaries, Edmondson examined Wordsworth’s famous poem, “Lines Composed a Few Miles from Tintern Abbey” written in 1798. Wordsworth’s life had become flat – “he lived in a din-filled city, among unfeeling people, and sensed that he is becoming one of them …there is a dull ache settling in his spirit” (p. 57). Returning to a scene from his childhood, he remembered himself as a young boy, free and reveling in nature. The return to nature, which is the heart of the poem, reminds him of its role in nurturing his own vitality. “Wordsworth’s poem enjoins us to feel that it (the answer to one’s despondency) lies somewhere within our reach – we are creatures who have the capacity to make ourselves sick, but also the power to heal ourselves” (p. 49).

Wordsworth’s poetry served the same vital function in the life of John Stuart Mill (1893/1989), who in early adulthood realized something deeply troubling – that he lacked the happiness central to the utilitarian philosophy in which he was immersed. Reflecting on his life, Mill described an early educational experience that was exceptional, but profoundly deficient. His father began teaching him Greek and Latin at a young age and then expanded the pedagogy to fields of philosophy, science, and mathematics. However, his father was deeply opposed to anything connected to sentiment or emotion. To escape the logic machine he had become, Mill began a quest to feel, and it was the poetry of Wordsworth, mostly about nature, that ministered deeply to the longings in his soul. He credited it for helping him recover from the crisis in his mental history.

To summarize, amidst the many interventions under study in PP, I lobby for a focus on encounters with nature, which some are already investigating. The preceding examples give us reason to believe that human lives may be enriched by such experiences. These can occur by being in nature as well as from reading about nature in poetry and literature, taking it in through film, or listening to music inspired by nature.

## Concluding Thoughts

My observations about what PP has accomplished over the last two decades are clearly mixed. Some may see the criticism as unfounded, if not mean-spirited, while others may view the input as long overdue straight talk about problems with an initiative intended to be transformational. I have long believed that self-criticism is central to making progress, whether in our individual lives, or our collective pursuits. My hope is thus that the field of PP will grow and flourish going forward, but also come to grips with its limitations. How might this happen?

One way is to pay attention to the problem of *overreach* in what PP claims to have accomplished. This will require greater scrutiny of the science touted as the evidential basis that PP works. Peer review is all we have to monitor the quality of the work that we do, but alas, it is an imperfect system, such that seriously flawed work sometimes gets published, even in high visibility outlets. There is the related problem of PP taking credit for more than it can credibly call its own achievements – i.e., the impact of PP (Rusk and Waters, 2013) has been overstated. As conveyed at the outset, extensive science on positive human functioning was happening well before PP declared its visionary new path. The upshot is that quantitative summaries of positive science unavoidably include many products that have nothing to do with the field of PP. Work from MIDUS is but one example of such wide-ranging science, much published in top-tier journals, showing protective benefits of psychological strengths. These studies were not created or nurtured by PP, and therefore, do not constitute evidence of its impact. Such distortion diminishes the stature of PP.

Relatedly there is need to recognize the *insularity* of PP, much seeming United States-centric, particularly in leadership. By creating its own professional society and journal, PP unfortunately removed itself from the wider discipline of psychology and its subfields, each with their own organizations and journals. While new groups can nurture comradery and a sense of identity, they can also create distance from related areas of inquiry. Most problematic, they can lead to insider peer reviewing that likely lowers rather than elevates the quality of the work generated.

On the matter of the *commercialization* of PP, I am perhaps an outlier in seeing this as a significant problem. However, it is construed, those who care about the long-term future of PP need to grapple with how to prevent the pursuit of profit from becoming a force that could ultimately take the enterprise down – on grounds that it is not scientifically substantiated, nor is it properly regulated, or doing lasting good, or is even creating harm. Without proper oversight, business pursuits could become the antithesis of the original promise and purpose of PP – to advance optimal human functioning.

Most of my essay has not been about these troublesome matters. Rather, I have tried to underscore the widespread consensus, from within PP and beyond, that thoughtful formulations are needed going forward, which put positives and negative together – i.e., research and practice that integrates human strengths and vulnerabilities. Parenthetically, one benefit of this shift may be that the adjective “positive” is less relentlessly present in titles of articles, books, and journals. As many have observed, greater attention must be given to diversity – i.e., how the wide array of topics being studied vary by numerous dimensions (e.g., age, gender, race/ethnicity, socioeconomic status, disability status, sexual orientation, and cultural context). It is also critical that societal relevance be a priority in the future science and practice that lies ahead. So doing demands attending to contemporary problems, and how they are negotiated in diverse life contexts. Our societal ills further call for study of negatives that have historically been neglected (greed, indifference, stupidity, and anger). Nonetheless, amidst the contemporary turbulence is the promise of the arts and of nature to help us be better – in seeing and caring about the suffering of others as well as in inspiring us to make the most of the lives we have been given and do so with commitment that encompasses families, schools, the workplace, communities, and the planet.

## Data Availability Statement

Publicly available datasets were analyzed in this study. This data can be found here: www.midus.wisc.edu.

## Author Contributions

The author confirms being the sole contributor of this work and has approved it for publication.

## Conflict of Interest

The author declares that the research was conducted in the absence of any commercial or financial relationships that could be construed as a potential conflict of interest.

## Publisher’s Note

All claims expressed in this article are solely those of the authors and do not necessarily represent those of their affiliated organizations, or those of the publisher, the editors and the reviewers. Any product that may be evaluated in this article, or claim that may be made by its manufacturer, is not guaranteed or endorsed by the publisher.

## Footnotes

1
www.midus.wisc.edu

2
www.authentichappiness.org

3
www.theflourishingcenter.com
